# Chronological Aging Standard Curves of Telomere Length and Mitochondrial DNA Copy Number in Twelve Tissues of C57BL/6 Male Mouse

**DOI:** 10.3390/cells8030247

**Published:** 2019-03-15

**Authors:** Ji Hyeong Baek, Hyeonwi Son, Young-Hoon Jeong, Sang Won Park, Hyun Joon Kim

**Affiliations:** 1 Department of Anatomy and Convergence Medical Sciences, Gyeongsang National University School of Medicine, Jinju 52727, Korea; baekbaek@gnu.ac.kr (J.H.B.); haeny@gnu.ac.kr (H.S.); 2 Institute of Health Sciences, Bio Anti-aging Medical Research Center, Gyeongsang National University, Jinju 52727, Korea; goodoctor@naver.com (Y.-H.J.); parksw@gnu.ac.kr (S.W.P.); 3 Department of Internal Medicine, Gyeongsang National University School of Medicine and Cardiovascular Center, Gyeongsang National University Changwon Hospital, Changwon 51472, Korea; 4 Department of Pharmacology and Convergence Medical Sciences, Gyeongsang National University School of Medicine, Jinju 52727, Korea

**Keywords:** telomere length, mitochondrial DNA copy number, aging, chronic stress, mouse

## Abstract

The changes in telomere length and mitochondrial DNA copy number (mtDNAcn) are considered to be aging markers. However, many studies have provided contradictory or only fragmentary information about changes of these markers in animal models, due to inaccurate analysis methods and a lack of objective aging standards. To establish chronological aging standards for these two markers, we analyzed telomere length and mtDNAcn in 12 tissues—leukocytes, prefrontal cortex, hippocampus, pituitary gland, adrenal gland, retina, aorta, liver, kidney, spleen, skeletal muscle, and skin—from a commonly used rodent model, C57BL/6 male mice aged 2–24 months. It was found that at least one of the markers changed age-dependently in all tissues. In the leukocytes, hippocampus, retina, and skeletal muscle, both markers changed age-dependently. As a practical application, the aging marker changes were analyzed after chronic immobilization stress (CIS) to see whether CIS accelerated aging or not. The degree of tissue-aging was calculated using each standard curve and found that CIS accelerated aging in a tissue-specific manner. Therefore, it is expected that researchers can use our standard curves to objectively estimate tissue-specific aging accelerating effects of experimental conditions for least 12 tissues in C57BL/6 male mice.

## 1. Introduction

Accumulating evidence indicates that telomere erosion and mitochondrial dysfunction contribute to cellular aging and aging-related diseases [1,2,3,4,5,6]. Telomeres are tandem repeats of non-coding sequences 5′-(TTAGGG)_n_-3′ at each chromosomal end, and are responsible for protecting the chromosome from sequence erosion and fusion with neighboring chromosomes. The telomere length of a human leukocyte is about 11 kb in the newborn and decreases to less than 4 kb in old age, not only due to the ‘end replication problem’ during repeated cell replication, but also by oxidative stress and inflammation [6,7,8]. Laboratory mice possess 50–150 kb telomeres [2]. Due to the triple-G-containing structure, the telomere sequence is sensitive to oxidative stress [9], alkylation [10], and ultraviolet (UV) irradiation [11]. Therefore, under chronic stress conditions, telomere shortening can also occur in adult neurons without DNA replication and active mitosis, by inducing telomeric double-strand breaks to a greater extent than in non-telomeric DNA [12]. Short dysfunctional telomeres can trigger tumor initiation by driving genomic instability, but they can also suppress cancer development by promoting cellular senescence or apoptosis pathways [3,13,14]. 

Mitochondrial DNA copy number (mtDNAcn) has been also documented to alter in aged tissues [1,4,5]. A critical aging regulator, mitochondria are multifunctional organelles that play an important role in cellular energy metabolism, reactive oxygen species generation, cell cycle regulation, cellular differentiation, and apoptosis [15]. Mitochondrial DNA (mtDNA) lacks histones and has a diminished DNA repair system to protect from and restore DNA damage. Therefore, it is more sensitive to oxidative stress than nuclear DNA, and mtDNA damage persists longer than nuclear DNA damage [16]. On the other hand, excessive mtDNAcn has been reported to result in nucleoid (an mtDNA–protein complex) enlargement and mitochondrial dysfunction [17]. Basal mtDNAcn ranges from 1000 to 10,000 per cell, and varies by organism, tissue, and cell type [18,19]. 

Telomere shortening and mtDNAcn alteration have correlations with oxidative stress and aging-related disorders such as diabetes, cardiovascular diseases, obesity, cancers, Alzheimer’s disease, and Parkinson’s disease, as well as psychiatric diseases [6,15,20,21,22,23]. However, many studies addressing telomere length and mtDNAcn changes in aged or diseased tissues have shown confusing and contradictory results [4,15,22]. Furthermore, although reliable alterations of telomere length and mtDNAcn by diseases or experimental treatments have been observed, it is not possible to quantify the effects on aging acceleration without chronological aging standard curves. 

Among various telomere length and mtDNAcn analysis methods, quantitative real-time polymerase chain reaction (qPCR) has recently become a preferred method. In this study, we provide age-dependent standard curves (2–24 months) of telomere length and mtDNAcn in 12 tissues from the C57BL/6 male mouse using qPCR. Based on slopes of these chronological standard curves, the aging acceleration effect of chronic stress was analyzed. 

## 2. Materials and Methods

### 2.1. Animals

Male C57BL/6 mice were housed in a specific pathogen-free-grade animal facility. Animals used for the standard curve included 10 mice each in the 2-, 6-, 12-month groups, 8 for the 18-month group, and 13 for the 24-month group. Tissues collected for the analysis were leukocytes, brain (prefrontal cortex and hippocampus), pituitary gland, adrenal gland, retina, thoracic aorta, liver, kidney, spleen, soleus skeletal muscle, and abdominal skin. 

### 2.2. Chronic Immobilization Stress

Chronic immobilization stress (CIS) was carried out as previously described [24,25,26]. Briefly, male 8-week-old mice were repeatedly placed in a restrainer for 2 h/day for 15 days after one-week habituation. Five control (CTL) and six stressed (STR) mice were sacrificed on the day after the final CIS. Animal use procedures were performed in accordance with the National Institute of Health (NIH) guidelines. The protocol (GLA-100917-M0093) was approved by the Gyeongsang National University Institution Animal Care & Use Committee (GNU IACUC). 

### 2.3. Telomere Length and mtDNAcn Analysis by Quantitative Real-Time PCR (qPCR)

Leukocytes were isolated from whole blood using erythrocyte lysis buffer (Qiagen, Hilden, Germany). Genomic DNA (gDNA) was extracted from leukocytes, whole blood, and tissues using the DNeasy Blood & Tissue Kit (Qiagen) or the Wizard SV 96 Genomic DNA Purification System (Promega, Madison, WI, USA) according to the manufacturer’s protocol. Primers for real-time PCR of telomeres, cytochrome c oxidase 1 and 2 (*Cox1* and *Cox2*), and a single-copy reference gene *36b4* were designed as previously reported (Table 1). 

The PCR amplification efficiencies of the primer sets were adjusted to be in the range of 1.9–2.1. The optimized PCR reaction mixture (10 μL, 384-well plate, triplicates per sample) contained the following components: 10 ng gDNA, 5 μL TopReal qPCR 2× PreMix (Enzynomics, Daejeon, Korea), sense and antisense primers (900 nM each for telomere, 500 nM each for *Cox1*, *Cox2*, and *36b4*). qPCR was performed using a LightCycler 480 II (Roche Molecular Diagnostics, Pleasanton, CA, USA). The optimized thermal program was one cycle at 95 °C for 10 min, two cycles at 95 °C for 15 s and 49 °C for 15 s for pre-amplification, followed by 45 cycles at 95 °C for 15 s, 62 °C for 10 s, and 72 °C for 15 s for telomere amplification. For *Cox1* and *Cox2*, the thermal program was one cycle at 95 °C for 10 min followed by 45 cycles at 95 °C for 15 s, 60 °C for 10 s, and 72 °C for 15 s. Amplification of *36b4* was performed in the same plates as the telomere or COXs. Following qPCR, the homogeneity of the PCR products was confirmed by melting curve analysis. The telomere length and mtDNAcn were calculated relative to the reference single-copy nuclear gene according to the 2^−ΔCt^ equation [30]: 2^(Cttar-Ct*36b4*)^ (Ct, threshold cycle; Ct_tar_, Ct value of targets, telomere, or *Cox2*; Ct*_36b4_*, Ct value of the reference single-copy gene, *36b4*).

### 2.4. Statistical Analysis

Data were presented as mean ± SEM. The D’Agostino–Pearson omnibus normality test followed by unpaired Student’s *t*-test was used to compare averages between two groups. These groups were CTL (*n* = 15) and STR (*n* = 18), which were formed from 3 technical replications of 5 CTL and 6 STR samples. To assess the reliability of linear regression lines of age standard curves, *r*^2^ (goodness of fit) and *p*-value (whether a slope is significantly non-zero) were shown in each graph. A *p*-value < 0.05 was considered statistically significant. Prism (ver. 5.01) (GraphPad, La Jolla, CA, USA) was used for all statistical analyses. 

## 3. Results

### 3.1. qPCR Condition Optimization

*Cox1* and *Cox2* PCR primers for the mtDNAcn analysis have gene-specific sequences. Therefore, PCR conditions for COX genes were determined simply by evaluating amplification efficiency and homogeneity of the PCR products via melting curve analysis (Figure S1). 

To determine a telomere qPCR protocol, various conditions were evaluated: primer sets, thermal programs, blood sample types, and qPCR master mixes. The thermal schedule for the telg and telc primer set in the original paper utilized a monochrome multiplex qPCR method [27]. In this study, as a modification, the reaction mixtures for telomere and the reference single-copy gene *36b4* were placed in different wells of the same PCR plate and the thermal cycler program was as described in the Materials and Methods section. PCR amplification efficiencies of telomere and *36b4* primer sets, and melting curves of their PCR products, were evaluated under the confirmed PCR conditions (Figure S1). Based on these preliminary tests, the telg–telc primer set and *Cox2* primer set were selected as major primers for this study.

The suitability of leukocytes and whole blood for the telomere length analysis were evaluated. Although leukocytes and whole blood samples were prepared from the same mice, the telomere length analysis using the leukocyte samples showed significant shortening of telomeres with aging, which was not observed in whole blood (Figure S2).

### 3.2. Mouse Age-Dependent Telomere Length and mtDNAcn Standard Curves

**Figure 1:**
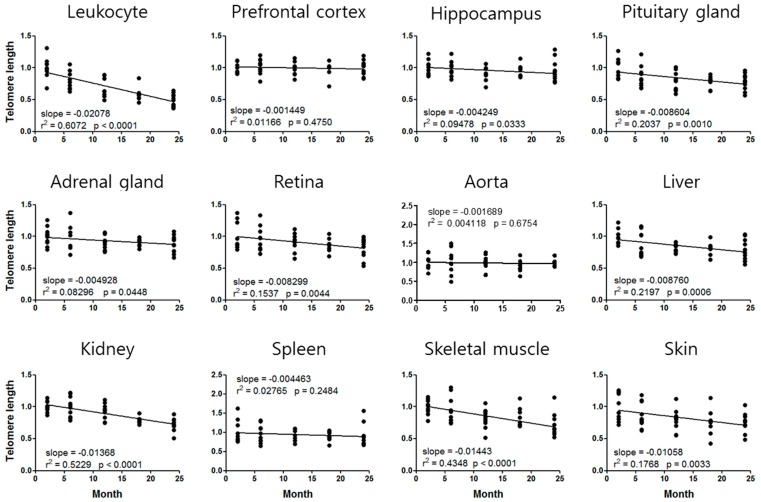
Aging standard curves for telomere length of C57BL/6 male mice. The relative telomere length at 2 months is normalized to 1. The slope of the linear regression line, goodness of fit (*r*^2^), and *p*-value (whether a slope is significantly non-zero) are provided for each curve.

Telomere length was significantly decreased in leukocytes, hippocampus, pituitary gland, retina, liver, kidney, skeletal muscle, and skin (Figure 1). The telomere length decline rate compared to the two-month-old was highest in the leukocytes (–50% at 24 months) and the skeletal muscle (–33% at 24 months) (Figure 1). 

**Figure 2:**
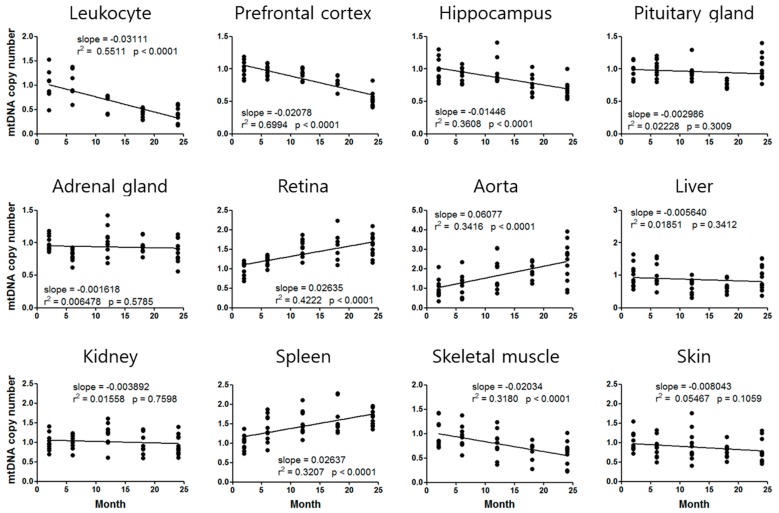
Aging standard curves for mitochondrial DNA copy number of C57BL/6 male mice. The relative mtDNAcn (analyzed by *Cox2*) at 2 months is normalized to 1. The slope of the linear regression line, goodness of fit (*r*^2^), and *p*-value (whether a slope is significantly non-zero) are provided for each curve.

The mtDNAcn exhibited an increasing or decreasing tendency depending on the tissues (Figure 2). The mtDNAcn of the leukocytes, prefrontal cortex, hippocampus, and soleus skeletal muscle decreased in an age-dependent manner, whereas those of the retina, thoracic aorta, and spleen increased with aging. The mtDNAcn fold change was highest in the leukocytes (2.4-fold higher at 2 months than at 24 months) and the thoracic aorta (2.4-fold higher at 24 months than at 2 months) (Figure 2). Analysis of mtDNAcn using *Cox1* also showed similar aging standard curves and slopes to those using *Cox2* (Figure S3).

### 3.3. Application of the Aging Standard Curves for Chronological Age Estimation

**Figure 3:**
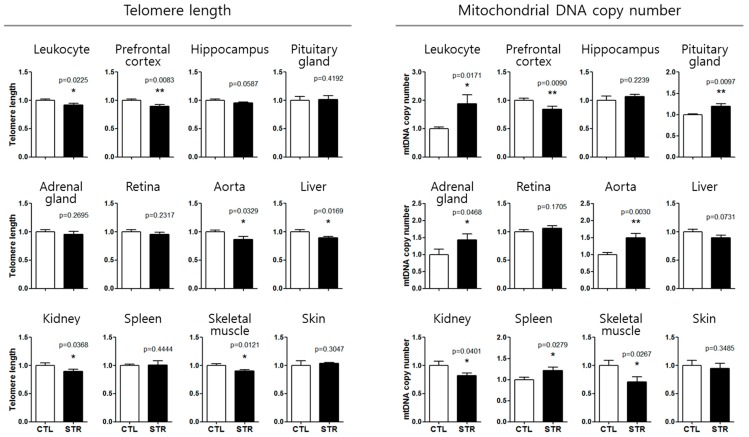
Chronic immobilization stress (CIS)-induced changes in telomere length and mitochondrial DNA copy number in 12 tissues. *, *p* < 0.05; **, *p* < 0.01 from unpaired Student’s *t*-test between control (CTL, *n* = 15) and stressed group (STR, *n* = 18).

As a practical application, we investigated telomere length and mtDNAcn alterations in 12 tissues of C57BL/6 male mice who had suffered from CIS that causes depression-like behaviors [24,25,26], and calculated telomere age and mtDNA age. Telomere length was significantly shortened by CIS in leukocytes, prefrontal cortex, aorta, liver, kidney, and skeletal muscle (Figure 3). The mtDNAcn was significantly decreased by CIS in the prefrontal cortex, kidney, and skeletal muscle, while it increased in the leukocytes, pituitary gland, adrenal gland, aorta, and spleen (Figure 3). The changes in mtDNAcn after CIS were also analyzed using *Cox1* primers, which were similar to those found using *Cox2* (Figure S4). 

Using slopes of the aging standard curves, telomere age and mtDNA age of the stressed mice were determined (Table 2). Aging degree was calculated when the markers were changed by both normal aging and CIS. Telomere age and mtDNA age after CIS were 6.3–14.3 months and 8.0–16.7 months, respectively, although the actual age was 2.5 months (10 weeks). This means that aging due to CIS was accelerated by 3.8–11.8 months in telomeres and by 5.5–14.2 months in mtDNA. 

The age of leukocyte mtDNA could not be calculated because the leukocyte mtDNAcn was increased by CIS, whereas its aging standard curve has a tendency to decrease (Figure 2). We also could not calculate the telomere age of the prefrontal cortex and aorta, and the mtDNA age of the pituitary gland, adrenal gland, and kidney, due to a lack of comparable age-dependent regression curves (Figure 1 and Figure 2). 

## 4. Discussion

Our study aimed to provide chronological aging standard curves and slopes of telomere length and mtDNAcn, which can help researchers objectively assess the degree of aging in target tissues in various studies using C57BL/6 male mice. C57BL/6 is one of the commonly used rodent models. Specifically, in psychiatric disorder studies, 67% of studies used male mice, and among them 35% used C57BL/6 male mice (searched in PubMed, on 9 February 2019). In addition, in the Jackson Lab list, 91% (1012 mice) of total live mouse products have C57BL/6 background (https://www.jax.org/). Therefore, the age standard curves of our study would be useful for many studies using C57BL/6 male mice.

To evaluate telomere length by qPCR, we used the telomere primer set telg and telc designed by Cawthon in 2009 [27]. Unlike previously suggested primers that generate PCR products of various lengths [29,31], the telg and telc set produced PCR products of constant length, resulting in stable amplification and clear chronological standard curves (Figure 1 and Figure S1). 

Telomere primer sequences were first suggested in 2002 (tel1 and tel2) [31], and then improved in 2009 (telg and telc) with higher accuracy [27]. However, the first primer set still has been cited much more than the new primer set. During 2017–2018, the 2002 paper was cited 515 times, whereas the 2009 paper was cited 227 times (Google Scholar, 12 November 2018). The 2009 paper suggested the monochrome multiplex qPCR method eliminates intra-assay variations due to pipetting errors. However, this method demands specific PCR software or machines, which can provide two different DNA amplification curves for one reaction well. This unusual demand limits universal use of the method. Therefore, we modified the protocol for universal use of the telg and telc primers in a monoplex method. 

It has been pointed out that as a telomere length analysis method, qPCR has a high variability [32,33]. However, the analysis results of qPCR using the telg–telc primer set have been reported to show good correlation with a standard method, telomere restriction fragment (TRF) analysis and the recently developed flow fluorescent in situ hybridization (flow FISH) [32,33]. Moreover, qPCR is rapid, efficient, economic, and applicable for all types of tissues from which gDNA could be extracted, which overcomes the limitations of TRF and flow FISH.

The telomere qPCR conditions proposed in this study resulted in reproducible and discriminating amplification outcomes, and the fidelity of the qPCR result was further confirmed by TRF analysis (Figure S5). The telomere standard curves also showed significant changes with aging. To the best of our knowledge, this is the first report of the aging standard curves of mouse telomeres using the telg and telc set and integrating various tissues across the body. 

All 12 tissues showed age-dependent changes in telomere length or mtDNAcn (Figure 1 and Figure 2), indicating that we can estimate tissue-specific aging status using at least one of these aging markers. A variety of studies have indicated that telomere erosion occurs in aged human or animal subjects [34]. In our study, all tissues showed telomere length decline with aging. However, the mtDNAcn showed a tendency to increase or decrease with aging depending on the tissue. We found increments in mtDNAcn in the retina, thoracic aorta, and spleen, but the other tissues showed a decreasing tendency with aging (Figure 2). 

In addition to mitochondrial dysfunction due to a decreased mitochondrial genome, increased mtDNAcn has also been suggested to be detrimental to cells and eventually induces cellular senescence or apoptosis [17]. Accumulation of mtDNA mutations induces high mtDNAcn in nucleoids (mtDNA–protein complexes), and results in nucleoid enlargement and subsequent mitochondria functional deficiency [35,36]. Excessive mtDNA replication could be triggered by the activation of twinkle mtDNA helicase and mitochondrial transcription factor A [17]. These previous studies support the notion that an increase in mtDNAcn is a normal phenomenon in aging, although the mechanism of tissue-specific increase or decrease with aging remains to be elucidated. 

We demonstrated the aging acceleration effect of CIS [24,25,26] by analyzing telomere length and mtDNAcn of 12 tissues. CIS significantly altered at least one of the two markers in 9 of 12 tissues, suggesting that CIS might accelerate aging of these tissues (Figure 3). Based on slopes of the aging standard curves in Figure 1 and Figure 2, we could calculate the degree of aging acceleration of each tissue by CIS (Table 2). These telomere length and mtDNAcn standard curves will provide a strategy for objective quantification of aging acceleration effects of experimental conditions in specific target tissues of the C57BL/6 male mouse, the most commonly used rodent model. 

Notably, telomere length of the prefrontal cortex and aorta, and mtDNAcn of the pituitary gland, adrenal gland, and kidney were changed by CIS, although they did not show significant age-dependent changes. Moreover, mtDNAcn of the leukocyte was increased by chronic stress, whereas it showed an age-dependent decreasing pattern (Figure 1, Figure 2 and Figure 3). These results suggest that CIS would be a serious risk factor for these tissues besides a simple aging accelerating effect. Various studies also demonstrated that mtDNAcn increased in the blood of patients with psychiatric disorder [15], cancer [37], lymphoma and leukemia [38], and mitochondrial depletion syndromes [39]. These results suggest that increased blood mtDNAcn by chronic stress could be associated with the development of neoplastic diseases or psychiatric disorders, including depression and anxiety, as shown in our previous studies [24,25,26]. 

Although there are few studies on telomere length or mtDNAcn changes in the prefrontal cortex due to stress, a post-mortem study in suicide completers showed short telomere length and lower mtDNAcn in the prefrontal cortex, as well as short telomere length and higher mtDNAcn in blood [40], which are similar to our findings in mice. Blood telomere length reduction and mtDNAcn increases have also been reported in patients with major depressive disorder, and in chronically stressed mice [41,42]. 

It is known that telomerase activity in adult tissues differs between human and rodents. Telomerase is constitutively expressed in various tissues of laboratory mice, whereas it is tightly regulated in human somatic cells [43]. Therefore, the results of mouse experiments cannot be directly applied to humans. Nevertheless, animal model experiments are indispensable to understanding human diseases, and the results have to be compared with human data to infer the clinical symptoms of the human body. 

In the present study, we investigated age-dependent changes of telomere length and mtDNAcn in 12 different mouse tissues and provided standard curves and slopes for telomere and mtDNA age calculation in each tissue. These results can be used to objectively estimate the aging accelerating effect of various experiments on each tissue, which will be helpful for many researchers in allowing them to make more convincing interpretations of their results regarding aging.

## Figures and Tables

**Table 1:** Primers used for quantitative real-time PCR.

Target	Name	Sequence	Ref.
Telomere	telg	5′-ACACTAAGGTTTGGGTTTGGGTTTGGGTTTGGGTTAGTGT-3′	[27]
(constant length)	telc	5′-TGTTAGGTATCCCTATCCCTATCCCTATCCCTATCCCTAACA-3′	
*Cox1*	*Cox1*-F	5′-CCCAGATATAGCATTCCCACG-3′	[28]
	*Cox1*-R	5′-ACTGTTCATCCTGTTCCTGC-3′	
*Cox2*	*Cox2*-F	5′-ATAACCGAGTCGTTCTGCCAAT-3′	[28]
	*Cox2*-R	5′-TTTCAGAGCATTGGCCATAGAA-3′	
*36b4*	*36b4*d	5′-ACTGGTCTAGGACCCGAGAAG-3′	[29]
	*36b4*d	5′-TCAATGGTGCCTCTGGAGATT-3′	

**Table 2:** Chronic immobilization stress (CIS)-induced aging acceleration. Telomere age and mitochondrial DNA age of stressed mice (STR) were calculated using the qPCR results (Figure 3) and the slope of the chronological aging standard curves (Figure 1 and Figure 2). The tissues that showed significant changes by both CIS and normal aging are presented.

**Telomere Length**	**STR (CTL = 1)**	**Slope (/Month)**	**Telomere Age** **(Actual Age = 2.5 months)**
**Leukocyte**	0.9204	−0.02078	6.3
**Liver**	0.8968	−0.00876	14.3
**Kidney**	0.8953	−0.01368	10.2
**Skeletal muscle**	0.9044	−0.01443	9.1
**mtDNAcn**	**STR (CTL = 1)**	**Slope (/Month)**	**mtDNA Age** **(Actual Age = 2.5 months)**
**Prefrontal cortex**	0.8460	−0.02780	8.0
**Aorta**	1.4990	0.06077	10.7
**Spleen**	1.2170	0.02637	10.7
**Skeletal muscle**	0.7116	−0.02034	16.7

## Supplementary Materials

The following are available online at https://www.mdpi.com/2073-4409/8/3/247/s1. Figure S1: Comparisons of qPCR primer sets for telomere length and mitochondrial DNA copy number analysis. (A–D) Standard curves and PCR efficiencies of standard gDNA (ranged from 1.48–40 ng) by each primer set. (E–G) Melting curve analysis of PCR products amplified by each primer set. Figure S2: Telomere length change analysis using purified leukocytes and whole blood of mice aged 2–18 months. The telg–telc primer set was used for the telomere amplification. **, *p* < 0.01; ***, *p* < 0.001 compared with the value at 2 months by one-way ANOVA with Dunnett’s multiple comparison test. Figure S3: Aging standard curves for mitochondrial DNA copy number (mtDNAcn) constructed by *Cox1* qPCR. The relative mtDNAcn at 2 months is normalized to 1. Slope of the linear regression line, goodness of fit (*r*^2^), and *p*-value (whether a slope is significantly non-zero) are provided for each curve. Figure S4: Chronic immobilization stress (CIS)-induced changes in mitochondrial DNA copy number in 12 tissues analyzed by *Cox1* qPCR. Error bar is SEM. *, *p* < 0.05; **, *p* < 0.01 from unpaired Student’s *t*-test. Figure S5: Telomere length analysis by a telomere restriction fragment (TRF) method. Telomere lengths of spleen and kidney were analyzed using TeloTAGGG Telomere Length Assay kit (Roche Diagnostics GmbH, Mannheim, Germany). Each 1.5 μg of gDNA was separated on 0.5% agarose gel and transferred to nylon membrane. The Digoxigenin-labeled telomere probe was hybridized and visualized by alkaline phosphatase-conjugated anti-DIG antibody. The mean TRF value was calculated according to the manufacturer’s protocol. CTL, pooled gDNA of 5 non-stressed control mice. STR, pooled gDNA of 6 chronically stressed mice. Relative telomere length by qPCR are the data in Figure 3 and Table 2.

## References

[1] Bratic A., Larsson N.G. (2013). The role of mitochondria in aging. J. Clin. Investig..

[2] Calado R.T., Dumitriu B. (2013). Telomere dynamics in mice and humans. Semin. Hematol..

[3] Deng Y., Chang S. (2007). Role of telomeres and telomerase in genomic instability, senescence and cancer. Lab. Investig..

[4] Kazachkova N., Ramos A., Santos C., Lima M. (2013). Mitochondrial DNA damage patterns and aging: Revising the evidences for humans and mice. Aging Dis..

[5] Lagouge M., Larsson N.G. (2013). The role of mitochondrial DNA mutations and free radicals in disease and ageing. J. Intern. Med..

[6] Rizvi S., Raza S.T., Mahdi F. (2014). Telomere length variations in aging and age-related diseases. Current Aging Sci..

[7] Arai Y., Martin-Ruiz C.M., Takayama M., Abe Y., Takebayashi T., Koyasu S., Suematsu M., Hirose N., von Zglinicki T. (2015). Inflammation, but not Telomere Length, Predicts Successful Ageing at Extreme Old Age: A Longitudinal Study of Semi-supercentenarians. EBioMedicine.

[8] Okuda K., Bardeguez A., Gardner J.P., Rodriguez P., Ganesh V., Kimura M., Skurnick J., Awad G., Aviv A. (2002). Telomere Length in the Newborn. Pediatr. Res..

[9] Henle E.S., Han Z., Tang N., Rai P., Luo Y., Linn S. (1999). Sequence-specific DNA cleavage by Fe^2+^-mediated fenton reactions has possible biological implications. J. Biol. Chem..

[10] Petersen S., Saretzki G., von Zglinicki T. (1998). Preferential accumulation of single-stranded regions in telomeres of human fibroblasts. Exp. Cell Res..

[11] Oikawa S., Tada-Oikawa S., Kawanishi S. (2001). Site-specific DNA damage at the GGG sequence by UVA involves acceleration of telomere shortening. Biochemistry.

[12] Wolkowitz O.M., Epel E.S., Reus V.I., Mellon S.H. (2010). Depression gets old fast: Do stress and depression accelerate cell aging?. Depress. Anxiety.

[13] Bernal A., Tusell L. (2018). Telomeres: Implications for Cancer Development. Int. J. Mol. Sci..

[14] Gilley D., Tanaka H., Herbert B.S. (2005). Telomere dysfunction in aging and cancer. Int. J. Biochem. Cell Biol..

[15] Picard M., McEwen B.S. (2018). Psychological Stress and Mitochondria: A Systematic Review. Psychosom. Med..

[16] Yakes F.M., Van Houten B. (1997). Mitochondrial DNA damage is more extensive and persists longer than nuclear DNA damage in human cells following oxidative stress. Proc. Natl. Acad. Sci. USA.

[17] Ylikallio E., Tyynismaa H., Tsutsui H., Ide T., Suomalainen A. (2010). High mitochondrial DNA copy number has detrimental effects in mice. Hum. Mol. Genet..

[18] Fuke S., Kubota-Sakashita M., Kasahara T., Shigeyoshi Y., Kato T. (2011). Regional variation in mitochondrial DNA copy number in mouse brain. Biochim. Biophys. Acta.

[19] Veltri K.L., Espiritu M., Singh G. (1990). Distinct genomic copy number in mitochondria of different mammalian organs. J. Cell. Physiol..

[20] Gilley D., Herbert B.S., Huda N., Tanaka H., Reed T. (2008). Factors impacting human telomere homeostasis and age-related disease. Mech. Ageing Dev..

[21] Hu L., Yao X., Shen Y. (2016). Altered mitochondrial DNA copy number contributes to human cancer risk: Evidence from an updated meta-analysis. Sci. Rep..

[22] Monroy-Jaramillo N., Dyukova E., Walss-Bass C. (2017). Telomere length in psychiatric disorders: Is it more than an ageing marker?. World J. Biol. Psychiatry.

[23] Zgheib N.K., Sleiman F., Nasreddine L., Nasrallah M., Nakhoul N., Isma’eel H., Tamim H. (2018). Short Telomere Length is Associated with Aging, Central Obesity, Poor Sleep and Hypertension in Lebanese Individuals. Aging Dis..

[24] Joo Y., Choi K.M., Lee Y.H., Kim G., Lee D.H., Roh G.S., Kang S.S., Cho G.J., Choi W.S., Kim H.J. (2009). Chronic immobilization stress induces anxiety- and depression-like behaviors and decreases transthyretin in the mouse cortex. Neurosci. Lett..

[25] Son H., Baek J.H., Go B.S., Jung D.H., Sontakke S.B., Chung H.J., Lee D.H., Roh G.S., Kang S.S., Cho G.J. (2018). Glutamine has antidepressive effects through increments of glutamate and glutamine levels and glutamatergic activity in the medial prefrontal cortex. Neuropharmacology.

[26] Son H., Jung S., Shin J.H., Kang M.J., Kim H.J. (2018). Anti-Stress and Anti-Depressive Effects of Spinach Extracts on a Chronic Stress-Induced Depression Mouse Model through Lowering Blood Corticosterone and Increasing Brain Glutamate and Glutamine Levels. J. Clin. Med..

[27] Cawthon R.M. (2009). Telomere length measurement by a novel monochrome multiplex quantitative PCR method. Nucleic Acids Res..

[28] Gomes A.P., Price N.L., Ling A.J., Moslehi J.J., Montgomery M.K., Rajman L., White J.P., Teodoro J.S., Wrann C.D., Hubbard B.P. (2013). Declining NAD^+^ induces a pseudohypoxic state disrupting nuclear-mitochondrial communication during aging. Cell.

[29] Callicott R.J., Womack J.E. (2006). Real-time PCR assay for measurement of mouse telomeres. Comp. Med..

[30] Pfaffl M.W. (2001). A new mathematical model for relative quantification in real-time RT-PCR. Nucleic Acids Res..

[31] Cawthon R.M. (2002). Telomere measurement by quantitative PCR. Nucleic Acids Res..

[32] Aubert G., Hills M., Lansdorp P.M. (2012). Telomere length measurement-caveats and a critical assessment of the available technologies and tools. Mutat. Res..

[33] Martin-Ruiz C.M., Baird D., Roger L., Boukamp P., Krunic D., Cawthon R., Dokter M.M., van der Harst P., Bekaert S., de Meyer T. (2015). Reproducibility of telomere length assessment: An international collaborative study. Int. J. Epidemiol..

[34] Jiang H., Ju Z., Rudolph K.L. (2007). Telomere shortening and ageing. Z. Gerontol. Geriatr..

[35] Bogenhagen D.F. (2010). Does mtDNA nucleoid organization impact aging?. Exp. Gerontol..

[36] Taylor R.W., Turnbull D.M. (2005). Mitochondrial DNA mutations in human disease. Nat. Rev. Genet..

[37] Chen N., Wen S., Sun X., Fang Q., Huang L., Liu S., Li W., Qiu M. (2016). Elevated Mitochondrial DNA Copy Number in Peripheral Blood and Tissue Predict the Opposite Outcome of Cancer: A Meta-Analysis. Sci. Rep..

[38] Lan Q., Lim U., Liu C.S., Weinstein S.J., Chanock S., Bonner M.R., Virtamo J., Albanes D., Rothman N. (2008). A prospective study of mitochondrial DNA copy number and risk of non-Hodgkin lymphoma. Blood.

[39] Xia C.Y., Liu Y., Yang H.R., Yang H.Y., Liu J.X., Ma Y.N., Qi Y. (2017). Reference Intervals of Mitochondrial DNA Copy Number in Peripheral Blood for Chinese Minors and Adults. Chin. Med. J. (Engl.).

[40] Otsuka I., Izumi T., Boku S., Kimura A., Zhang Y., Mouri K., Okazaki S., Shiroiwa K., Takahashi M., Ueno Y. (2017). Aberrant telomere length and mitochondrial DNA copy number in suicide completers. Sci. Rep..

[41] Cai N., Chang S., Li Y., Li Q., Hu J., Liang J., Song L., Kretzschmar W., Gan X., Nicod J. (2015). Molecular Signatures of Major Depression. Curr. Biol..

[42] Lin P.Y., Huang Y.C., Hung C.F. (2016). Shortened telomere length in patients with depression: A meta-analytic study. J. Psychiatr. Res..

[43] Prowse K.R., Greider C.W. (1995). Developmental and tissue-specific regulation of mouse telomerase and telomere length. Proc. Natl. Acad. Sci. USA.

