# Supplementary material for: Chronological Aging Standard Curves of Telomere Length and Mitochondrial DNA Copy Number in Twelve Tissues of C57BL/6 Male Mouse
Source: Cells. 2019 Mar 15;8(3):247. doi: 10.3390/cells8030247 (PMC6468494; doi:10.3390/cells8030247)
Supplement: Supplementary file 1 [file cells-08-00247-s001.pdf]

Supplementary Figure 1

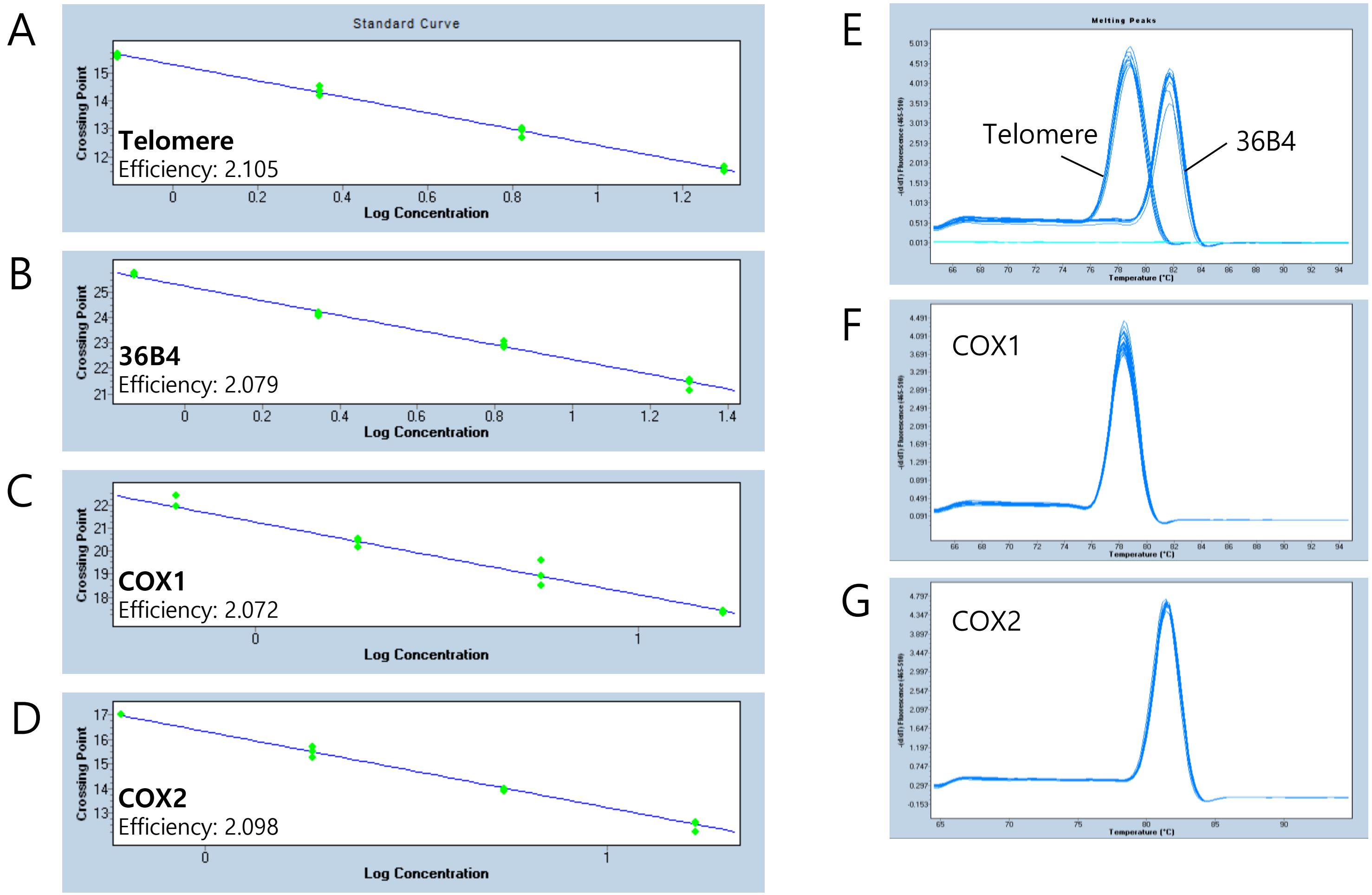

Supplementary Figure 2

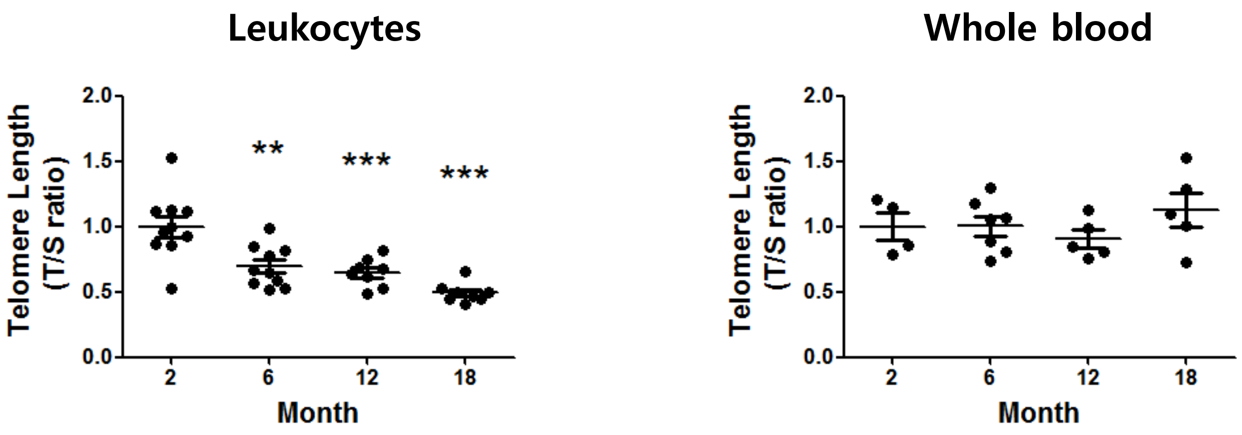

Supplementary Figure 3 – mtDNAcn by COX1

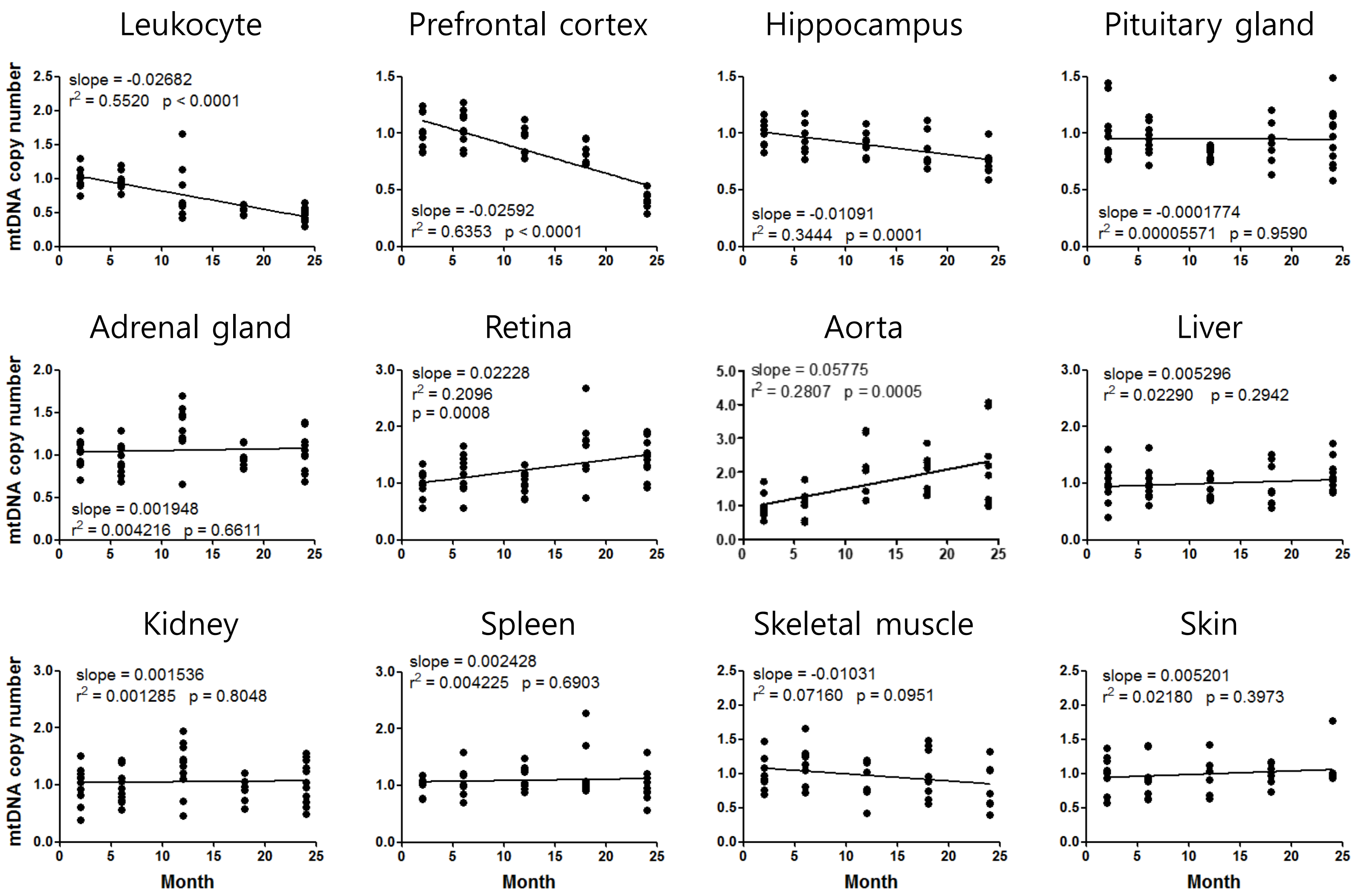

Supplementary Figure 4 – CIS mouse mtDNAcn by COX1

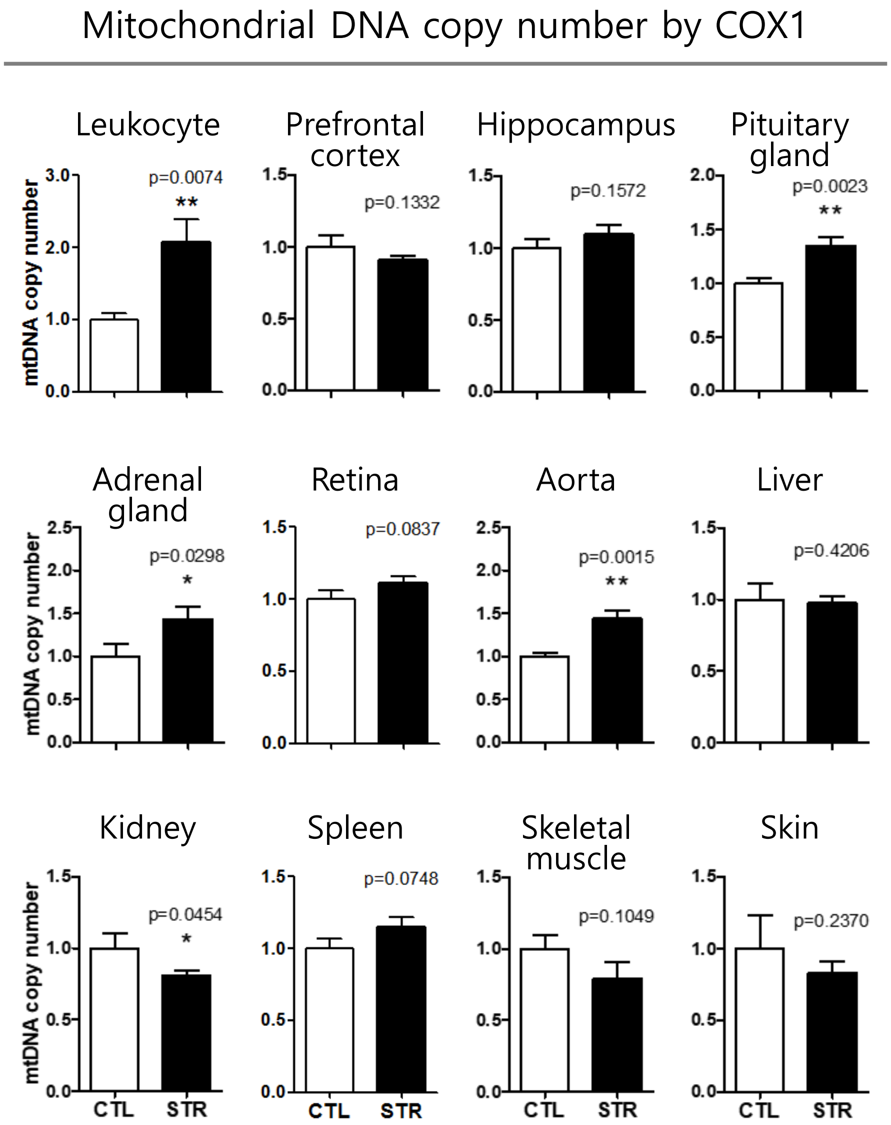

Supplementary Figure 5

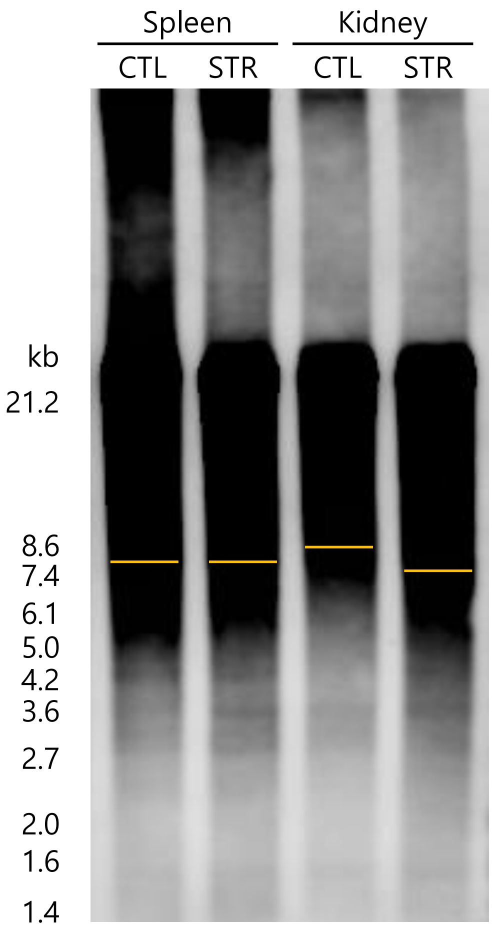

Mean TRF (kb)	7.7	7.7	8.2	7.4
Normalized	1	0.9952	1	0.9016
qPCR	1	1.0012	1	0.8953
